# Effects of Asparagus Powder Supplementation on Glycemic Control, Lipid Profile, and Oxidative Stress in Overweight and Obese Adults: An Exploratory Randomized Controlled Trial

**DOI:** 10.3390/life15101584

**Published:** 2025-10-10

**Authors:** Jittima Mongraykang, Tadsawiya Padkao, Orachorn Boonla, Yothin Teethaisong, Thapanee Roengrit, Sukrisd Koowattanatianchai, Piyapong Prasertsri

**Affiliations:** 1 National Institute of Health, Department of Medical Sciences, Ministry of Public Health, Nonthaburi 11000, Thailand; plaijittima2540@gmail.com (J.M.); 2 Faculty of Allied Health Sciences, Burapha University, Chonburi 20131, Thailandorachorn@go.buu.ac.th (O.B.); yothin.te@go.buu.ac.th (Y.T.); 3 Institute of Medicine, Suranaree University of Technology, Nakhon Ratchasima 30000, Thailand; thapanee.ro@sut.ac.th (T.R.); 4 Division of Cardiology, Department of Medicine, Burapha Hospital, Burapha University, Chonburi 20131, Thailand; sukrisd.ko@buu.ac.th (S.K.)

**Keywords:** antioxidant, diabetes, obesity, phytochemical nutrient

## Abstract

This study investigated the effects of asparagus powder supplementation on blood glucose regulation, insulin, lipid profile, and oxidative stress in overweight and obese individuals. Forty-four adults aged 18–59 years participated in a 12-week randomized controlled trial and were randomly assigned to receive either asparagus powder (40 mg/kg/day) or a placebo (maltodextrin, 40 mg/kg/day). Assessments included an oral glucose tolerance test (OGTT), fasting blood glucose (FBG), insulin, homeostasis model assessment of insulin resistance (HOMA-IR) and β-cell function (HOMA-B), lipid profile, and oxidative stress markers (malondialdehyde [MDA], protein carbonyl, and superoxide dismutase [SOD]). In the asparagus group, OGTT at 30 min and low-density lipoprotein cholesterol (LDL-C) significantly decreased, while SOD activity significantly increased (all *p* < 0.05). In contrast, the placebo group showed significant increases in OGTT at 30 min, insulin, HOMA-IR, HOMA-B, triglycerides (TG), the TG/high-density lipoprotein cholesterol (HDL-C) ratio, and the total cholesterol (TC)/HDL-C ratio (all *p* < 0.05). Between-group comparisons indicated that FBG, area under the BG curve at 30–120 min, TG, TG/HDL-C, and MDA levels were significantly lower in the asparagus group than in the placebo group (all *p* < 0.05), whereas OGTT, LDL-C, SOD activity, insulin, HOMA-IR, HOMA-B, and TC/HDL-C did not differ significantly. Other indices, including TC, HDL-C, and protein carbonyl, showed no significant within- or between-group differences. In conclusion, 12 weeks of asparagus powder supplementation partially improved glycemic control, lipid profile, and oxidative stress in overweight and obese individuals. These findings suggest a potential role of asparagus as a complementary nutritional strategy to reduce the risk of diabetes and cardiovascular disease in this population.

## 1. Introduction

Obesity is one of the most prevalent chronic diseases and represents a major global health challenge. Its prevalence has increased substantially at both the global and regional levels. In 2021, an estimated 1.00 billion adult males and 1.11 billion adult females were classified as overweight or obese [1]. The underlying cause is abnormal or excessive fat accumulation that exceeds metabolic requirements. This excess adiposity contributes to numerous complications through anatomical and metabolic pathways, elevating the risk of morbidity and mortality from conditions such as type 2 diabetes mellitus, cardiovascular disease, coronary heart disease, stroke, and certain cancers [2].

Beyond excessive food intake and insufficient physical activity, another widely recognized contributor to overweight and obesity is the consumption of high-fat diets, which disrupt gut microbiota and lead to intestinal dysbiosis [3]. Gut microbiota plays a critical role in maintaining health by regulating several mechanisms, including the suppression of fasting-induced adipose factor expression in the intestine [4]. Dysfunction of gut microbiota impairs fat metabolism, enhances fat accumulation, promotes inflammation, elevates blood glucose and insulin levels, increases free radical production, and contributes to insulin resistance [5].

Numerous studies have highlighted the association between obesity and type 2 diabetes, which is mechanistically linked to insulin resistance through elevated levels of non-esterified fatty acids, glycerol, hormones, and pro-inflammatory cytokines [6]. In addition, higher oxidative stress markers, such as plasma malondialdehyde (MDA) and protein carbonyl, have been observed in overweight and obese individuals [7,8]. Concurrently, reductions in antioxidant defenses—including superoxide dismutase (SOD) and vitamins A, C, and E—have also been reported in these populations [7,9]. Therefore, alongside lifestyle modifications such as an appropriate diet and regular physical activity, the use of nutraceuticals or dietary supplements may represent an adjunct strategy to prevent or delay the progression to type 2 diabetes [10].

Asparagus (*Asparagus officinalis* L.) is widely regarded as a health-promoting food due to its high fiber content, low calorie density, and rich phytochemical profile, including saponins, flavonoids, phenolics, vitamins, fructans, and cinnamic acids [11]. These bioactive compounds have been recognized for their pharmacological properties, such as antidiabetic, anticancer, antioxidant, hypolipidemic, and antimicrobial effects [12]. In addition, asparagus provides key nutritional components—including dietary fiber, inulin, low-molecular-weight carbohydrates, low-molecular-weight polyphenols, and macromolecular polyphenols—that contribute to its prebiotic effects. Through these mechanisms, asparagus may exert beneficial influences on host health and gut microbiota when incorporated into a healthy diet [13]. Notably, prebiotic substances such as soluble dietary fiber and inulin have been shown to reduce glucose absorption across the intestinal lining and decrease fat absorption, thereby supporting improvements in blood glucose and lipid regulation [14].

Despite these well-recognized benefits, evidence regarding the effects of asparagus supplementation on obesity and overweight remains limited. Therefore, the present study aimed to investigate the potential benefits of asparagus supplementation on blood glucose, insulin, homeostasis model assessment of insulin resistance (HOMA-IR) and β-cell function (HOMA-B), lipid profile, and oxidative stress in overweight and obese individuals. In addition, body composition and fat distribution were evaluated to provide further insight into the impact of supplementation.

## 2. Materials and Methods

### 2.1. Participants and Sample Size

This exploratory randomized controlled trial was conducted between 20 December 2022 and 14 September 2023 in overweight and obese male and female individuals aged 18–59 years residing in Chonburi Province, Thailand.

The sample size was determined using the standard formula for comparing two means in an experimental study, based on the findings of Nishimura et al. [15], who administered 6 g/day of asparagus bottom-stem extract for 10 weeks. In that study, mean fasting blood glucose (FBG) decreased from 104.1 ± 3.7 mg/dL to 100.9 ± 3.4 mg/dL. Using an effect size (ΔFBG) of 3.2 mg/dL, with α = 0.05 and β = 0.10 (power = 90%), the required sample size was estimated at 20 participants per group. To account for an anticipated 20% dropout rate, the target enrollment was increased to 24 participants per group, resulting in a total of 48 participants. The calculation was based on expected changes in FBG, which was designated as the primary endpoint. In contrast, body weight and body composition were considered exploratory outcomes and were not incorporated into the sample size calculation; therefore, findings related to weight outcomes should be considered preliminary.

### 2.2. Ethical Consideration

All participants provided written informed consent after receiving both verbal and written information about this study. The study protocol was reviewed and approved by the Human Ethics Committee of Burapha University (IRB3-115/2565; approval date: 20 December 2022) and was registered at ClinicalTrials.gov (ID: NCT06195813; registration date: 10 January 2024).

### 2.3. Screening of Participants

Participants underwent health screenings that included assessment of vital signs, medical history, and health questionnaires. In addition, a three-day dietary intake record and physical activity records were collected. The inclusion criteria were as follows: (a) male or female, aged 18–59 years; (b) body mass index (BMI) ≥ 23 kg/m^2^, according to the Asia-Pacific classification; (c) absence of chronic or infectious diseases, including diabetes, cardiovascular disease, liver disease, kidney disease, endocrine disorders, neurological disorders, toxic thyroid disease, or other infectious conditions; and (d) non-smoker and not a regular alcohol consumer. The exclusion criteria were as follows: (a) regular consumption of supplements (e.g., vitamins) or medications that could influence study outcomes; (b) professional athletes or individuals engaged in regular physical exercise routines; (c) history of food allergies, particularly to shoot-type vegetables such as asparagus; (d) female participants who were pregnant, breastfeeding, or planning pregnancy within three months; and (e) presence of infection-related symptoms (e.g., fever) or a positive COVID-19 antigen test.

### 2.4. Experimental Protocols

#### 2.4.1. Baseline Measurements

**Figure 1:**
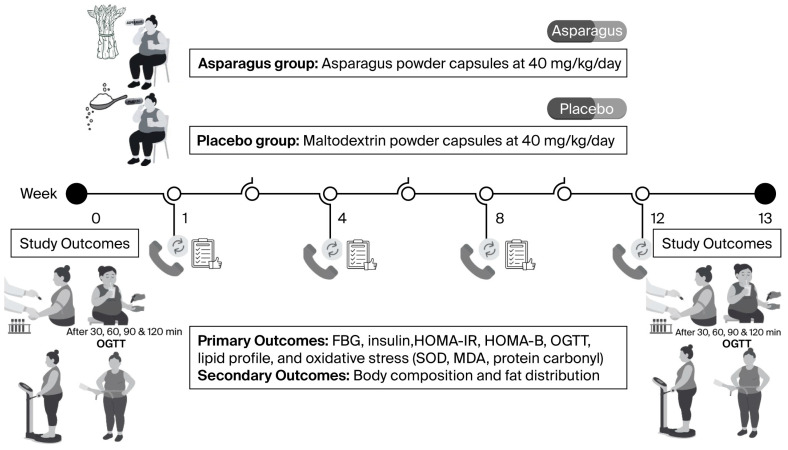
Experimental protocol of the study, illustrating the timeline of screening, baseline assessments, randomization, supplementation, and post-test measurements.

Approximately one week after screening, participants reported to the laboratory and were asked to rest for 5–10 min. Thereafter, vital signs, body composition, and fat distribution were measured. Blood samples (approximately 10 mL) were collected to assess FBG, insulin, lipid profile, and oxidative stress biomarkers (Figure 1).

Following blood collection, an oral glucose tolerance test (OGTT) was conducted. Participants consumed a glucose solution containing 75 g of glucose powder dissolved in 100 mL of drinking water. BG levels were then measured from fingertip samples at 30, 60, 90, and 120 min post ingestion.

#### 2.4.2. Randomization and Supplementation

**Figure 2:**
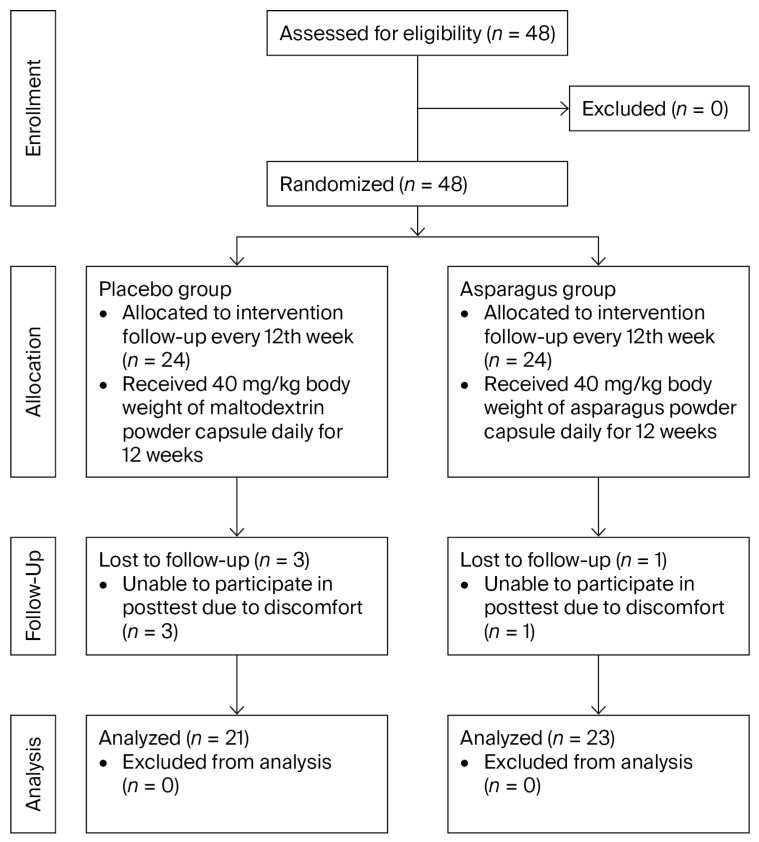
CONSORT flow diagram of participant enrollment, allocation, follow-up, and analysis.

A flow diagram of the study is presented in Figure 2. After baseline measurements, participants were randomly assigned to either the placebo group (*n* = 24) or the asparagus group (*n* = 24) using simple random allocation via IBM SPSS Statistics version 25. Allocation concealment was ensured through central randomization, with a designated researcher (P.P. and S.K.) generating the sequence and assigning participants. Blinding was maintained throughout the trial: participants, interventionists (J.M.), data collectors (T.P.), outcome assessors (O.B.), and data analysts (T.R. and Y.T.) were all blinded to group allocation.

Previous studies by Ahmad et al. [16] demonstrated that asparagus extract at 200 mg/kg body weight significantly reduced lipopolysaccharide-induced oxidative stress in rats. To translate this dose to humans, body surface area normalization was applied [17] using the following formula:

Human equivalent dose (mg/kg) = Animal dose (mg/kg) × Animal Km factor (rat = 6)/Human Km factor (adult = 37).

Based on this calculation, the equivalent human dose was 200 × (6/37) = 32.43 mg/kg. For practical purposes, this was rounded to 40 mg/kg, corresponding to 500 mg per capsule. Participants in the asparagus group consumed capsules at this dosage (e.g., ~2 g/day for a 50 kg individual), with each capsule containing 500 mg and a total daily intake of 5–7 capsules (2.5–3.5 g/day) over 12 weeks. The placebo group received maltodextrin capsules of equivalent weight and appearance. If the calculated dosage was not evenly divisible by the capsule content, adjustments were made using rounding principles (rounding up if the excess exceeded 250 mg and rounding down if below 250 mg). For example, a participant weighing 70 kg would be assigned six capsules per day.

For comparison, Nishimura et al. [15] administered asparagus powder at 6 g/day (120 mg/kg/day for a 50 kg individual), approximately twice the dose used in the present study. In contrast, the current trial aimed to investigate the minimum effective dose of asparagus powder in overweight and obese adults. The selected dose of 40 mg/kg was further supported by preclinical evidence of hypoglycemic and lipid-lowering effects at equivalent levels when scaled to human body weight. For adults weighing 50–60 kg, this corresponds to ~2.0–2.5 g/day, which lies within the range of customary vegetable intake and below thresholds associated with gastrointestinal intolerance. Accordingly, this dosage was considered biologically plausible and safe.

Participants were instructed on how to consume and store the capsules at home. The daily dose was divided into two intakes (e.g., three capsules after breakfast and three capsules after dinner). Capsules were to be stored in a cool, dry place, avoiding refrigeration. Participants were also asked to record supplement intake daily. During the intervention, they were instructed to maintain their usual lifestyle, including physical activity and dietary habits. Weekly follow-ups were conducted by phone or via the Line application to monitor compliance. Adherence was assessed using the pill count method and categorized as high (≥80%) or low (<80%).

Participants were instructed to immediately report any adverse events potentially related to supplementation, including allergic reactions (e.g., skin rash and oral swelling), gastrointestinal symptoms (e.g., nausea and diarrhea with blood), or respiratory symptoms (e.g., nasal congestion, coughing, and difficulty breathing). They were also asked to notify the research team if they received medical treatment or experienced changes in medication or vitamin use that could influence study outcomes.

#### 2.4.3. Post-Supplementation Measurements

At the end of the 12-week intervention, all measurements were repeated following the same procedures as those used at baseline. Prior to testing, participants were instructed to obtain at least seven hours of sleep on the night before the post-test. Upon arrival at the laboratory, participants rested for 5–10 min before undergoing sequential assessments of vital signs, body composition, fat distribution, blood sampling, and OGTT. All measurements were performed under standardized environmental and physiological conditions to minimize variability.

Participants who experienced serious adverse effects related to supplementation, exhibited poor adherence (<80%), withdrew consent, or were unable to complete the post-test were excluded from the final analysis.

### 2.5. Preparation of Asparagus Powder and Placebo

The asparagus powder used in this study was manufactured by Rai Sai Chol 101 Co., Ltd., Phetchabun, Thailand, a company certified by the Thai Food and Drug Administration (FDA; License No. 6720236220006) and operating under Good Manufacturing Practice (GMP) standards [18]. Fresh green asparagus spears were thoroughly washed with tap water, thinly sliced, and dried in a hot-air oven at 60 °C for 12–14 h until a final moisture content of 9–10% was reached. The dried spears were then finely ground into powder and encapsulated. Each capsule contained 500 mg of asparagus powder.

The placebo capsules were formulated to closely resemble the asparagus capsules in color and appearance, with each capsule containing 500 mg of maltodextrin. Encapsulation of both asparagus powder and maltodextrin was performed by Phanat Nikhom Hospital, Chonburi, Thailand, under controlled conditions (22–28 °C and relative humidity < 60%) in accordance with SOP-PHANAT guidelines. Quality control procedures included monitoring capsule weight deviation (<10%) and moisture content (<10%). The asparagus powder was not standardized for specific bioactive compounds, and formal stability testing was not conducted; these aspects are acknowledged as limitations of the present trial.

### 2.6. Analysis of Asparagus Powder Constituents

The phytochemical constituents of asparagus powder were analyzed using gas chromatography–mass spectrometry (GC–MS). An Agilent 7890A gas chromatograph coupled with an Agilent 7000B mass spectrometer (Agilent Technologies, Santa Clara, CA, USA) equipped with an HP-5 capillary column (30 m × 0.32 mm i.d., 0.25 µm film thickness) was employed. Briefly, 100 mg of asparagus powder was dissolved in 1 mL of ethanol, and a 2 µL aliquot was injected in split mode (5:1) using an autosampler. The injection temperature was 250 °C, with helium as the carrier gas at a flow rate of 1.0 mL/min. The oven program was initiated at 40 °C (held for 5 min), increased to 200 °C over 25 min, and then ramped to 280 °C over 61 min. The mass spectrometer was operated in electron ionization mode (70 eV), with an ion source temperature of 230 °C and a scan range of 50–650 m/z. Compound identification was performed using GC-QQQ software (2020) in conjunction with the NIST MS Search 2.0 library (National Institute of Standards and Technology, Gaithersburg, MD, USA).

GC–MS analysis identified several volatile compounds and fatty acids, including hexadecanoic acid, propionic acid, pyrone derivatives, dodecyl acrylate, stearic acid, and ferulic acid. It should be noted that GC–MS primarily detects volatile and fatty acid constituents and does not quantify non-volatile bioactives such as inulin-type fructans, saponins, and flavonoids. In addition, propionic acid, a short-chain fatty acid detected Via GC–MS, is distinct from indole-3-propionic acid, a tryptophan-derived microbial metabolite with different biological functions. From the GC–MS analysis, the major constituents of asparagus powder were hexadecanoic acid (20.21%), propionic acid (7.17%), and pyrone (3.47%). The additional phytochemicals identified included dodecyl acrylate (9.15%), pentadecanoic acid (0.55%), tetradecanoic acid (0.79%), octadecanoic acid (1.46%), 4-cyclopentene-1,3-dione (0.45%), 3,7,11,15-tetramethyl-2-hexadecen-1-ol (0.91%), propanoic acid (0.80%), acetic acid (0.18%), and furfural (3.25%).

### 2.7. Biochemical Assays

Following an overnight fast of at least 8 h, approximately 10 mL of venous blood was collected from each participant and analyzed for FBG, insulin, and lipid profile—including total cholesterol (TC), triglycerides (TG), low-density lipoprotein cholesterol (LDL-C), and high-density lipoprotein cholesterol (HDL-C)—according to standard clinical laboratory protocols (National Healthcare Systems Co., Ltd., Chonburi, Thailand). Ratios of TC/HDL-C, TG/HDL-C, and LDL-C/HDL-C were subsequently calculated.

HOMA indices were applied to evaluate diabetes risk, as they are widely used in epidemiologic studies. Elevated HOMA-IR and reduced HOMA-B are consistently associated with increased diabetes risk [19]. The following formulas were used [20]: HOMA-IR = [fasting insulin (µU/mL) × FBG (mg/dL)]/405; HOMA-B = [20 × fasting insulin (µU/mL)]/[FBG (mg/dL) − 63].

An OGTT was conducted by administering a glucose solution containing 75 g of glucose dissolved in 100 mL of drinking water. BG concentrations were determined from fingertip samples at 30, 60, 90, and 120 min post ingestion. The area under the curve (AUC) for BG was then calculated.

### 2.8. Oxidative Stress Assays

Plasma malondialdehyde (MDA, µM), a biomarker of lipid peroxidation, was measured in ethylenediaminetetraacetic acid-treated samples using high-performance liquid chromatography with fluorescence detection, following a previously described method [21] with minor modifications. Plasma protein carbonyl (nmol/mg protein), a biomarker of protein oxidation, was determined using a colorimetric assay based on the derivatization of carbonyl groups with 2,4-dinitrophenylhydrazine, according to a previously reported protocol [22] with modifications. In addition, serum superoxide dismutase (SOD) activity (% inhibition), an indicator of antioxidant defense, was measured using a colorimetric method with the SOD Assay Kit-WST (Dojindo Laboratories, Kumamoto, Japan) following the manufacturer’s instructions.

### 2.9. Body Composition and Fat Distribution Measurements

BM and height were measured using a standard weight and height scale (Health o 268 meter Pro Series, McCook, IL, USA), and BMI was subsequently calculated. Waist and hip circumferences were assessed using a standard measuring tape, and the waist/hip ratio (W/H ratio) was derived [23]. Body composition parameters—including body fat percentage, fat-free mass, muscle mass, body water, protein mass, mineral mass, visceral fat level, basal metabolic rate, and fitness score—were evaluated using a bioelectrical impedance analyzer (InBody270, InBody Co., Ltd., Daejeon, Republic of Korea) [24].

### 2.10. Data Analyses

Data distribution and homogeneity of variance were assessed using the Shapiro–Wilk test and Levene’s test, respectively. Between-group differences at baseline were analyzed using independent *t*-tests. Differences within and between groups over time were examined using two-way repeated-measures analysis of variance (ANOVA) with age adjustment, followed by Bonferroni correction. Where appropriate, paired *t*-tests were conducted to confirm within-group changes. For all relevant outcomes, partial eta-squared (η^2^) was reported as the measure of effect size, along with corresponding F-values and *p*-values. Statistical analyses were performed using SPSS Statistics (IBM Corp., Armonk, NY, USA), with statistical significance set at *p* < 0.05.

## 3. Results

### 3.1. Baseline Participant Characteristics

Of the 48 participants initially enrolled, four withdrew from the study because they were unable to complete the post-test (*n* = 1 in the asparagus group; *n* = 3 in the placebo group). Accordingly, 44 participants completed the trial, comprising 23 in the asparagus group (96%) and 21 in the placebo group (88%), and their data were included in the final analysis. Supplementation adherence was high in both groups, with a rate of 86% in the asparagus group and 84% in the placebo group.

The majority of participants were female (*n* = 38; 86.36%), while six were male (13.64%). At baseline, no significant differences were observed between groups in physical and physiological characteristics—including age, sex distribution, height, BM, BMI, basal metabolic rate, fitness score, systolic and diastolic blood pressure (SBP and DBP), pulse rate, respiratory rate, body temperature, and hemoglobin saturation (all *p* > 0.05) (Table 1). Although age did not differ significantly between the placebo and asparagus groups (24.52 ± 5.98 vs. 21.78 ± 4.08 years), it was considered a potential numerical difference that could confound the outcomes; therefore, age was included as a covariate in all analyses of primary and secondary outcomes.

Additionally, analysis of three-day physical activity and dietary intake records, collected either at baseline or during the 12-week supplementation period, showed no significant between-group differences (all *p* > 0.05) (Table 1).

### 3.2. Body Composition and Fat Distribution

In the placebo group, both BM (*p* = 0.034) and BMI (*p* = 0.034) increased significantly after supplementation, whereas no significant changes were observed in the asparagus group (BM: *p* = 0.221; BMI: *p* = 0.285). The W/H ratio significantly decreased following asparagus supplementation (*p* = 0.045) but remained unchanged in the placebo group (*p* = 0.536). However, between-group comparisons showed no significant differences in BM (partial η^2^ = 0.021, F(1, 41) = 0.881, *p* = 0.353), BMI (partial η^2^ = 0.010, F(1, 41) = 0.428, *p* = 0.517), or W/H ratio (partial η^2^ = 0.029, F(1, 41) = 1.245, *p* = 0.271).

Other parameters—including waist circumference, hip circumference, body fat percentage, fat-free mass, muscle mass, body water, protein mass, mineral mass, and visceral fat level—did not differ significantly within or between groups (all *p* > 0.05) (Table 1).

### 3.3. FBG and Insulin Concentrations, HOMA-IR, and HOMA-B

After 12 weeks of supplementation, FBG concentration did not change significantly in either the placebo group (*p* = 0.313) or the asparagus group (*p* = 0.262). In the placebo group, insulin concentration, HOMA-IR, and HOMA-B significantly increased (*p* = 0.001, *p* = 0.001, and *p* = 0.005, respectively), whereas no significant changes were observed in the asparagus group (*p* = 0.189, *p* = 0.309, and *p* = 0.275, respectively).

**Figure 3:**
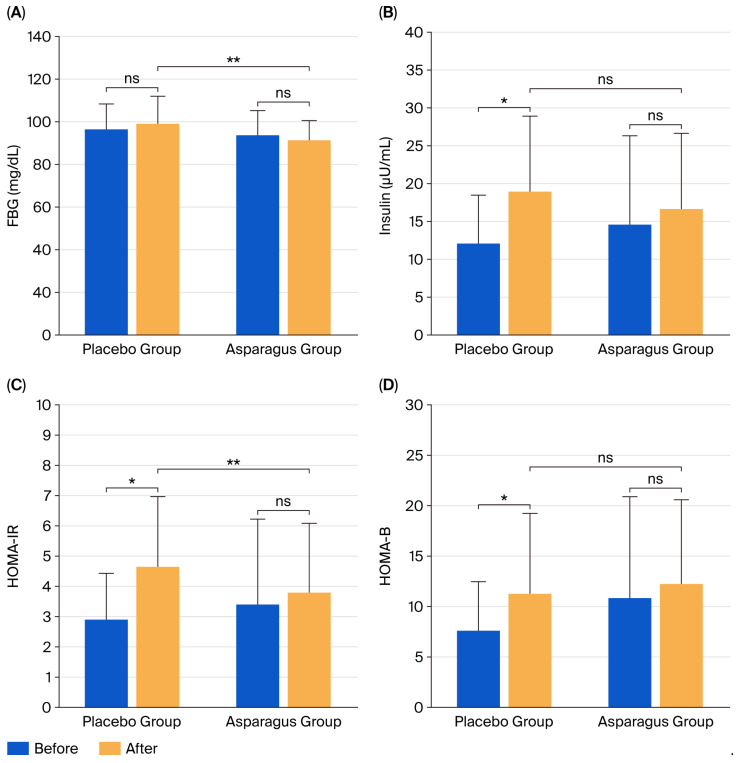
Fasting blood glucose (FBG) (**A**), insulin (**B**), homeostasis model assessment of insulin resistance (HOMA-IR) (**C**), and β-cell function (HOMA-B) (**D**) in participants from the placebo and asparagus groups at baseline and after 12 weeks of supplementation. Values are expressed as mean ± SD. Statistical analyses were performed using two-way repeated measures ANOVA with Bonferroni Post Hoc tests. * *p* < 0.05 vs. baseline; ** *p* < 0.05 vs. placebo group; ns, not statistically significant (*p* > 0.05).

Between-group comparisons showed no significant differences in insulin concentration (partial η^2^ = 0.030, F(1, 41) = 1.248, *p* = 0.271), HOMA-IR (partial η^2^ = 0.053, F(1, 41) = 2.232, *p* = 0.143), or HOMA-B (partial η^2^ = 0.000, F(1, 41) = 0.004, *p* = 0.950). In contrast, FBG concentration was significantly lower in the asparagus group compared to the placebo group (partial η^2^ = 0.089, F(1, 41) = 3.903, *p* = 0.045) (Figure 3).

### 3.4. Blood Glucose Concentration in Response to OGTT

**Figure 4:**
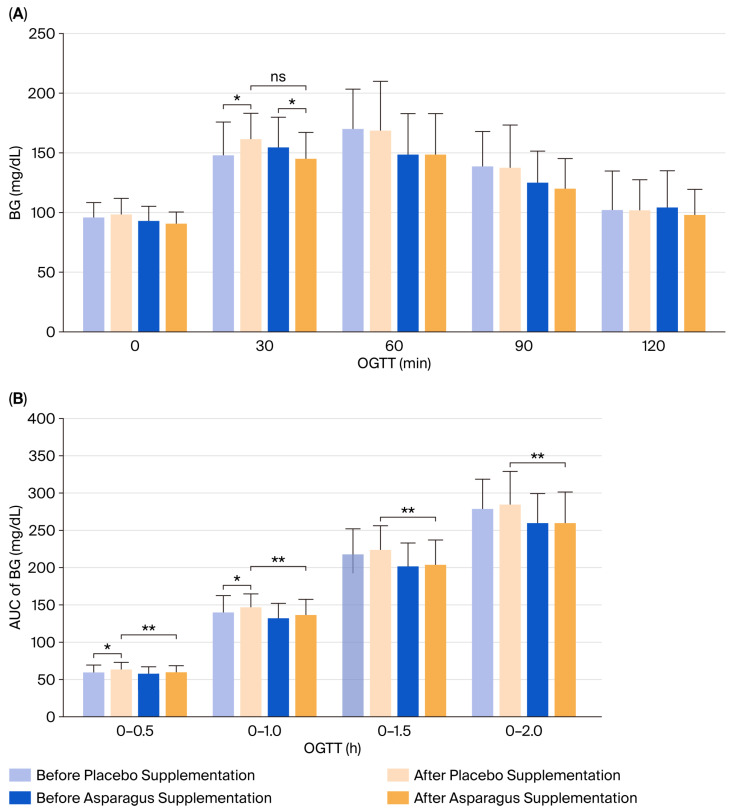
Blood glucose (BG) responses to the oral glucose tolerance test (OGTT) (**A**) and area under the curve (AUC) of BG (**B**) in participants from the placebo and asparagus groups at baseline and after 12 weeks of supplementation. Values are expressed as mean ± SD. * *p* < 0.05 vs. baseline; ** *p* < 0.05 vs. placebo group; ns, not statistically significant (*p* > 0.05).

In response to the OGTT, BG concentration at 30 min significantly increased in the placebo group after 12 weeks of supplementation (*p* = 0.013), whereas it significantly decreased in the asparagus group (*p* = 0.043). However, the between-group comparison revealed no significant difference (partial η^2^ = 0.015, F(1, 41) = 0.616, *p* = 0.437) (Figure 4). No significant within-group changes were observed at 60, 90, or 120 min (all *p* > 0.05), and no significant between-group differences were detected across these time points (all *p* > 0.05).

Furthermore, AUC values for 0–30 min (*p* = 0.009) and 0–60 min (*p* = 0.036) significantly increased in the placebo group following the intervention, whereas no such changes were observed in the asparagus group (all *p* > 0.05). Between-group comparisons revealed that post-supplementation AUCs at 0–30, 0–60, 0–90, and 0–120 min were significantly lower in the asparagus group compared to the placebo group (AUC0–30: partial η^2^ = 0.057, F(1, 41) = 2.433, *p* = 0.047; AUC0–60: partial η^2^ = 0.060, F(1, 41) = 2.573, *p* = 0.047; AUC0–90: partial η^2^ = 0.073, F(1, 41) = 3.146, *p* = 0.044; AUC0–120: partial η^2^ = 0.068, F(1, 41) = 2.930, *p* = 0.045) (Figure 4).

### 3.5. Lipid Profile

After the supplementation period, TG levels (*p* = 0.045), the TG/HDL-C ratio (*p* = 0.041), and the TC/HDL-C ratio (*p* = 0.049) were significantly increased in the placebo group. In contrast, LDL-C levels were significantly decreased in the asparagus group (*p* = 0.014).

**Figure 5:**
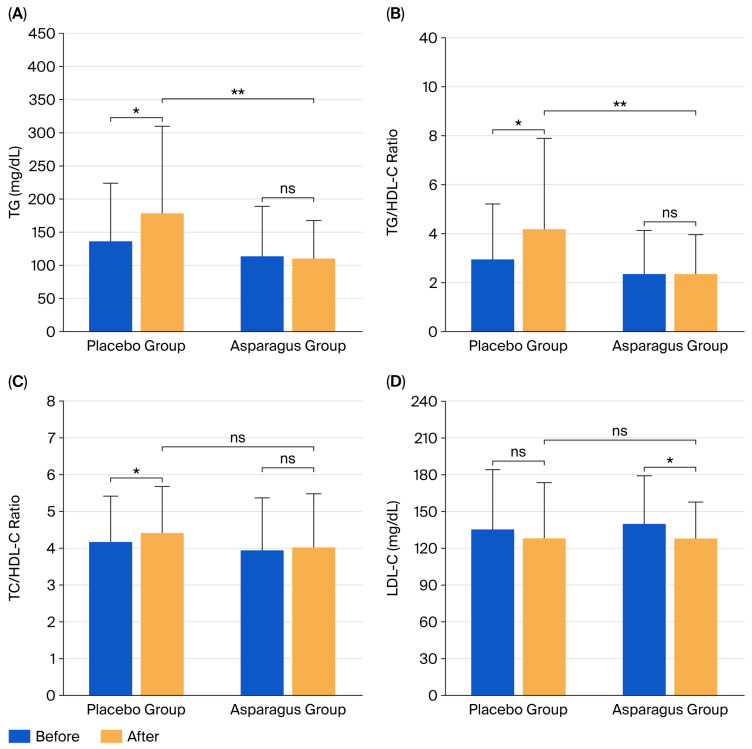
Triglyceride (TG) levels (**A**), TG/high-density lipoprotein cholesterol (HDL-C) ratio (**B**), total cholesterol (TC)/HDL-C ratio (**C**), and low-density lipoprotein cholesterol (LDL-C) (**D**) in participants from the placebo and asparagus groups at baseline and after 12 weeks of supplementation. Values are expressed as mean ± SD. * *p* < 0.05 vs. baseline; ** *p* < 0.05 vs. placebo group; ns, not statistically significant (*p* > 0.05).

Between-group analyses revealed that TG concentration (partial η^2^ = 0.065, F(1, 41) = 2.777, *p* = 0.043) and the TG/HDL-C ratio (partial η^2^ = 0.056, F(1, 41) = 2.366, *p* = 0.032) were significantly lower in the asparagus group compared to the placebo group. In contrast, no significant between-group differences were found for the TC/HDL-C ratio (partial η^2^ = 0.012, F(1, 41) = 0.466, *p* = 0.499) or LDL-C levels (partial η^2^ = 0.000, F(1, 41) = 0.019, *p* = 0.891) (Figure 5). Furthermore, no significant within- or between-group differences were observed for TC levels (partial η^2^ = 0.006, F(1, 41) = 0.261, *p* = 0.612), HDL-C levels (partial η^2^ = 0.010, F(1, 41) = 0.386, *p* = 0.538), or the LDL-C/HDL-C ratio (partial η^2^ = 0.003, F(1, 41) = 0.107, *p* = 0.746) (Table 2).

### 3.6. Oxidative Stress

After 12 weeks of supplementation, MDA levels did not differ significantly from baseline in either the asparagus group (*p* = 0.207) or the placebo group (*p* = 0.953). Similarly, protein carbonyl concentrations showed no significant changes within groups (asparagus: *p* = 0.459; placebo: *p* = 0.920). In contrast, SOD activity significantly increased in the asparagus group (*p* = 0.024), whereas no significant change was observed in the placebo group (*p* = 0.087).

**Figure 6:**
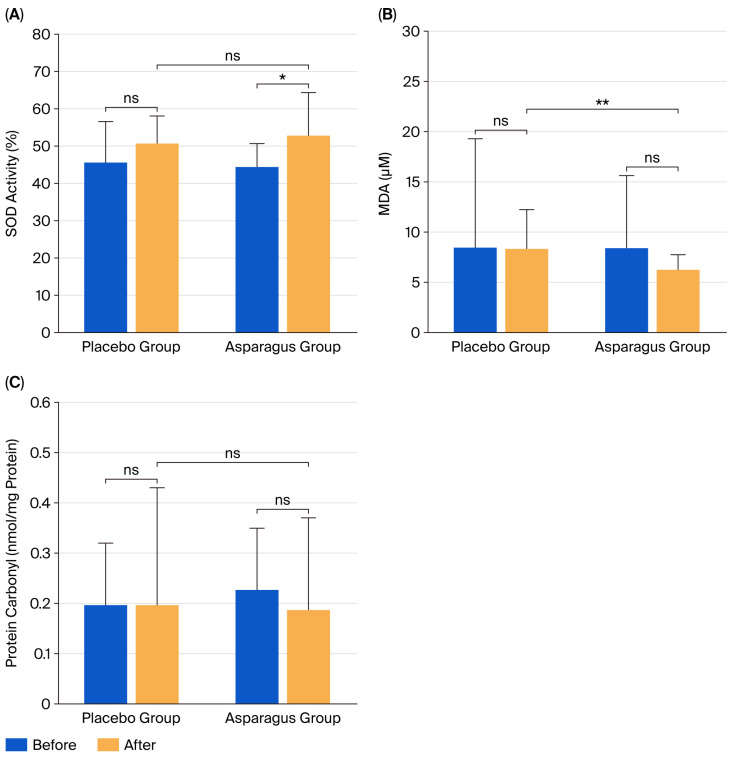
Superoxide dismutase (SOD) activity (**A**), malondialdehyde (MDA) (**B**), and protein carbonyl (**C**) levels in participants from the placebo and asparagus groups at baseline and after 12 weeks of supplementation. Values are expressed as mean ± SD. * *p* < 0.05 vs. baseline; ** *p* < 0.05 vs. placebo group; ns, not statistically significant (*p* > 0.05).

Between-group comparisons showed that MDA levels were significantly lower in the asparagus group compared to the placebo group (partial η^2^ = 0.160, F(1, 41) = 7.626, *p* = 0.009) (Figure 6). In contrast, no significant between-group differences were observed for protein carbonyl (partial η^2^ = 0.008, F(1, 41) = 0.295, *p* = 0.590) or SOD activity (partial η^2^ = 0.016, F(1, 41) = 0.567, *p* = 0.457).

## 4. Discussion

In this study, asparagus supplementation at a dose of 40 mg/kg body weight per day for 12 weeks led to significantly lower FBG, AUC for BG, TG, TG/HDL-C ratio, and MDA levels compared to placebo. Other metabolic and antioxidant indices—including OGTT, LDL-C, SOD activity, insulin, HOMA-IR, HOMA-B, and TC/HDL-C ratio—showed within-group changes but did not differ significantly between groups. Similarly, body composition indices (BM, BMI, and W/H ratio) changed within groups but were not significantly different between the asparagus and placebo groups.

Participants demonstrated high adherence to supplementation, with adherence rates of 86% in the asparagus group and 84% in the placebo group. Consistent with this, both groups exhibited changes from baseline; however, the directions of change diverged. The asparagus group showed partial improvements in glycemic control, lipid profile, and antioxidant capacity, suggesting a beneficial effect of asparagus supplementation. In contrast, the placebo group demonstrated unfavorable changes, including deterioration in glycemic control and lipid profile.

Specifically, participants in the placebo group experienced significant increases in BM and BMI after the supplementation period. Weight gain is influenced by multiple factors, most notably dietary intake. Although dietary intake did not change significantly in this group, it gradually exceeded energy expenditure from physical activity. The resulting energy surplus was metabolized and stored as triacylglycerol in adipose tissue, contributing to increased body weight and waist circumference [25]. In parallel, adverse metabolic changes were observed, including elevated BG levels at 30 min during the OGTT, along with higher insulin, HOMA-IR, HOMA-B, and lipid indices (TG, TG/HDL-C ratio, and TC/HDL-C ratio).

From a pathophysiological standpoint, the expansion of adipose tissue can precipitate metabolic dysfunction characterized by hyperglycemia, dyslipidemia, inflammation, oxidative stress, and insulin resistance [26]. Mechanistically, adipocytes secrete adipokines that regulate energy balance, triacylglycerol storage and mobilization, and insulin signaling in glucose and lipid metabolism. Dysregulated adipokine secretion promotes insulin resistance in skeletal muscle by reducing glucose uptake, inhibiting glucose oxidation, and suppressing hepatic glycogen storage, thereby elevating BG levels. Moreover, adipokines exert divergent effects on hepatic glucose metabolism: adiponectin and leptin suppress gluconeogenesis, while resistin promotes gluconeogenesis and glycogenolysis. Inflammatory mediators such as interleukin-6 (IL-6) decrease glycogen synthesis, and tumor necrosis factor-α (TNF-α) disrupts hepatic insulin signaling by impairing insulin receptor pathways [27]. In addition, increased free fatty acids from lipolysis, together with lipid intermediates (e.g., long-chain fatty acyl-CoAs, diacylglycerol, and ceramides), activate serine-phosphorylating enzymes that inhibit insulin receptor function. Collectively, these mechanisms contribute to the development and progression of insulin resistance [28].

It is also noteworthy that participants in the placebo group consumed maltodextrin at 40 mg/kg body weight (~2 g/day), which may have contributed to the significant increases in insulin, HOMA-IR, HOMA-B, and TG levels observed in this group. These findings highlight the importance of recognizing that maltodextrin, although commonly used as a control carbohydrate in nutrition studies, is not physiologically inert. As a rapidly digestible polysaccharide, maltodextrin can elevate postprandial glucose and insulin levels, thereby influencing outcomes that might otherwise be attributed to the experimental intervention [29]. Furthermore, emerging evidence suggests that maltodextrin can modulate gut microbiota composition and function. For example, certain strains of *E. coli* and *Clostridium* utilize maltodextrin as a substrate, potentially leading to microbial imbalance, impaired barrier function, and heightened inflammatory responses in the host [30]. A recent study further demonstrated that supplementation with 10 g/day of maltodextrin significantly reduced gut microbiota diversity [31].

In contrast, participants in the asparagus group demonstrated a reduction in the W/H ratio. Furthermore, key outcomes improved following 12 weeks of asparagus powder supplementation, including decreases in BG levels in response to OGTT at 30 min and LDL-C, alongside an increase in SOD activity. Our findings are consistent with previous studies. Nishimura et al. [15] reported that asparagus powder intake significantly lowered FBG levels compared to placebo. Specifically, supplementation with 6 g/day of asparagus powder for 10 weeks reduced FBG from 104.1 mg/dL to 100.9 mg/dL. Moreover, their study also demonstrated reductions in TC, SBP, DBP, and the cardio-ankle vascular index score. In diabetic rat models, 21 consecutive days of asparagus extract supplementation significantly reduced FBG, OGTT values at 30, 60, and 120 min, AUC for BG, and TG levels [32]. Similarly, supplementation with asparagus extract at 250 and 500 mg/kg/day for 28 days significantly reduced BG levels and increased both insulin concentrations and the insulin-to-glucose ratio [33]. Taken together, the present findings expand current evidence on the BG-lowering effects of asparagus supplementation, demonstrating its beneficial impact in obese and overweight individuals.

With respect to blood lipid levels, a significant reduction in LDL-C was observed after 12 weeks of asparagus supplementation. This finding is consistent with several previous studies suggesting that asparagus extract exerts cholesterol-lowering effects. In hyperlipidemic mice, supplementation with *n*-butanol asparagus extract at doses of 40, 80, or 160 mg/kg body weight for 8 weeks significantly reduced body weight gain, TC, and LDL-C levels, while increasing HDL-C levels [34]. Similarly, García et al. [35] reported hypocholesterolemic and hepatoprotective effects of freeze-dried asparagus in hypercholesterolemic rats. In their study, daily supplementation with 125, 250, or 500 mg/kg body weight for 5 weeks led to significant reductions in TC and LDL-C levels, particularly at the 250 and 500 mg/kg doses. Collectively, these findings—including those from the present study—support the pharmacological potential of asparagus in effectively reducing blood lipid levels and improving lipid metabolism.

We also observed a BP-lowering effect of asparagus supplementation, reflected in a significant reduction in DBP from baseline by approximately 4.2 mmHg. This result is consistent with the findings of Nishimura et al. [15], who reported significant decreases in SBP (~8.6 mmHg) and DBP (~4.6–5.4 mmHg) in healthy volunteers following asparagus supplementation. Similarly, Sanae and Yasuo [36] demonstrated a significant reduction in SBP in spontaneously hypertensive rats fed a diet containing 5% asparagus for 10 weeks. Together, these findings suggest that asparagus may confer additional cardiovascular benefits, not only by improving lipid metabolism but also by reducing BP, thereby contributing to a lower risk of cardiovascular disease [37].

Furthermore, results from our GC–MS analysis identified propionic acid, a short-chain fatty acid, as one of the predominant constituents of asparagus powder. Propionic acid has been recognized as an important mediator in the interplay between diet, gut microbiota, and host physiology. It exerts several pharmacological effects, including reducing hepatic and plasma fatty acid levels, decreasing food intake, exerting immunosuppressive activity, and potentially improving tissue insulin sensitivity [38]. Accordingly, propionic acid may have contributed to the observed benefits in this study and should be considered a promising bioactive compound in the context of obesity and type 2 diabetes prevention.

In addition, asparagus supplementation for 12 weeks significantly increased SOD activity, although the change did not differ significantly from the placebo group. Elevated SOD activity is considered beneficial to health, as SOD plays a pivotal role in the antioxidant defense system by neutralizing reactive oxygen species (ROS), regulating the activity of other ROS-scavenging enzymes, and preventing oxidative stress, which is often elevated in individuals with obesity [39]. Supporting evidence comes from experimental studies. Ho et al. [40] demonstrated that treatment with asparagus stem extract (5 mg/mL for 6 h) significantly decreased ROS generation and DNA damage while increasing glutathione (GSH) synthesis in bovine cumulus–granulosa cells. In mice exposed to lead toxicity, supplementation with asparagus aqueous extract (400 mg/kg orally for 14 days) improved oxidative stress biomarkers (lipid peroxidation, nitric oxide, GSH levels, and activities of SOD, catalase, glutathione peroxidase, and glutathione reductase), inflammation markers (IL-1β, TNF-α, NF-κB, and iNOS), and apoptosis-related proteins (caspase-3 activity, Bax, Bcl-2, and proliferating cell nuclear antigen) [41]. Moreover, in rats treated with bisphenol A, a known inducer of organ toxicity, co-administration of asparagus extract (400 mg/kg body weight/day for 8 weeks) significantly decreased MDA levels and increased both GSH and total antioxidant capacity. However, supplementation with asparagus extract alone did not significantly affect serum antioxidant status [42].

In addition to benefits related to obesity and type 2 diabetes prevention, propionic acid has demonstrated positive effects on immunocompetence, antioxidant defense, and anti-inflammatory processes [43,44]. Furthermore, our GC–MS analysis revealed that hexadecanoic acid and pyrone were also abundant constituents of asparagus powder. These compounds are known to exert multiple biological activities, including antioxidant, anti-inflammatory, anticancer, antimicrobial, and antiviral effects [45,46]. Notably, hexadecanoic acid has been reported to possess antioxidant properties and to contribute to the prevention of atherosclerosis in animal models [46]. The presence of propionic acid, hexadecanoic acid, and pyrone therefore provides a mechanistic basis for the therapeutic potential of asparagus supplementation in enhancing antioxidant capacity.

Despite these findings, MDA and protein carbonyl concentrations were not significantly reduced following asparagus supplementation, although they showed a downward trend (MDA: −2.14 µM, −24.97%; protein carbonyl: −0.04 nmol/mg protein, −17.15%). This suggests that while the phytochemical content of the asparagus powder used in this study was sufficient to enhance antioxidant enzyme activity (e.g., SOD), it may not have been high enough to significantly suppress the oxidative by-products of lipid and protein oxidation. Another possible explanation is that oxidative stress levels in our obese and overweight participants were not as elevated as in other populations studied previously, since our participants were otherwise healthy and free from chronic diseases.

To the best of our knowledge, this is the first study to evaluate the metabolic and antioxidant benefits of asparagus supplementation in an overweight and obese population. Nonetheless, several limitations and directions for future research should be considered. Standardization of asparagus powder through detailed compositional analyses and formal stability testing is essential. In this study, GC–MS detected volatile compounds and fatty acids but did not capture inulin-type fructans, saponins, or flavonoids—bioactive constituents known to influence glycemic control, lipid metabolism, and antioxidant activity. Future trials should therefore incorporate quantitative analyses of dietary fiber, inulin, saponins, and flavonoids using advanced methods such as HPLC or LC–MS/MS. Mechanistic assessments should also be expanded to include adipokines, cytokines, gut microbiota, short-chain fatty acids, and appetite-regulating hormones to clarify underlying pathways. Participant attrition (one in the asparagus group and three in the placebo group) may have reduced statistical power, particularly for outcomes related to metabolic parameters, antioxidant markers, and body composition, and the small sample size precluded formal adjustment for multiplicity. Longer intervention periods (≥6–12 months) with adequately powered samples are recommended, especially when adiposity is a primary endpoint, to more effectively evaluate the durability of weight and metabolic benefits. Body weight and composition should be pre-specified as primary outcomes when appropriate, and intention-to-treat analyses with appropriate handling of missing data should be applied. Supplementation adherence was assessed only by pill count, a method prone to bias; thus, multimethod adherence monitoring (e.g., electronic packaging, clinician assessments, and self-reports) is advisable. Finally, dose-finding trials and studies in broader clinical populations, including individuals with metabolic syndrome, prediabetes, and type 2 diabetes, are warranted to enhance generalizability and therapeutic potential. Reporting effect sizes with confidence intervals will further improve interpretability and reproducibility across studies.

## 5. Conclusions

This randomized controlled trial demonstrated that 12 weeks of asparagus powder supplementation provided modest metabolic and antioxidant benefits in overweight and obese individuals, partially improving blood glucose regulation, lipid profile, and oxidative stress. From a nutritional therapy standpoint, incorporating asparagus into dietary plans may serve as a complementary strategy for individuals at risk of obesity or type 2 diabetes. Such an approach could help support blood sugar and lipid control, thereby contributing to a reduced risk of cardiometabolic diseases. Outcomes related to body weight should be considered exploratory. Further standardized, long-term trials are warranted to confirm these findings and to establish asparagus powder as a reproducible nutritional therapy for metabolic health.

## Figures and Tables

**Table 1:** Physical and physiological characteristics of participants in the placebo and asparagus groups at baseline and after 12 weeks of supplementation.

Parameters	Placebo Group (*n* = 21)			Asparagus Group (*n* = 23)			*p*-Value (After vs. After)
	Before	After	Δ Change (%)	Before	After	Δ Change (%)	
Age (years)	24.52 ± 5.98	-	-	21.78 ± 4.08	-	-	0.081
Sex (male/female) (n, %)	2/19 (10/90)	-	-	4/19 (17/83)	-	-	0.459
Height (cm)	161.62 ± 5.63	-	-	160.91 ± 7.53	-	-	0.729
Body mass (kg)	74.81 ± 9.93	75.85 ± 9.17 *	1.03 (1.38)	71.53 ± 9.55	72.47 ± 9.86	0.93 (1.30)	0.353
Body mass index (kg/m^2^)	28.68 ± 4.01	29.09 ± 3.66 *	0.41 (1.42)	27.74 ± 4.34	28.06 ± 4.07	0.32 (1.16)	0.517
Body fat (%)	40.77 ± 5.37	41.47 ± 5.53	0.70 (1.71)	37.75 ± 7.51	38.92 ± 7.39	1.17 (3.10)	0.200
Muscle mass (kg)	24.47 ± 3.49	24.41 ± 3.48	−0.06 (−0.23)	24.24 ± 4.18	24.18 ± 4.33	−0.06 (−0.25)	0.937
Fat-free mass (kg)	31.28 ± 6.59	31.72 ± 6.69	0.44 (1.42)	27.71 ± 7.76	28.37 ± 7.44	0.66 (2.38)	0.155
Water mass (kg)	32.53 ± 4.14	32.51 ± 4.18	−0.02 (−0.07)	32.65 ± 5.06	32.29 ± 5.23	−0.36 (−1.09)	0.919
Protein mass (kg)	8.77 ± 1.14	8.75 ± 1.16	−0.02 (−0.22)	8.71 ± 1.38	8.68 ± 1.42	−0.03 (−0.30)	0.920
Mineral mass (kg)	3.13 ± 0.33	3.13 ± 0.32	0.00 (−0.02)	3.12 ± 0.47	3.13 ± 0.52	0.01 (0.21)	0.911
Waist circumference (cm)	94.17 ± 10.57	94.62 ± 10.53	0.45 (0.48)	89.89 ± 11.18	89.76 ± 11.37	−0.13 (−0.15)	0.216
Hip circumference (cm)	107.31 ± 6.89	108.19 ± 6.79	0.88 (0.82)	104.20 ± 13.65	103.76 ± 13.51	−0.43 (−0.42)	0.305
Waist/hip ratio	0.88 ± 0.09	0.88 ± 0.10	0.00 (−0.24)	0.87 ± 0.10	0.86 ± 0.10 *	−0.01 (−0.51)	0.271
Visceral fat level	14.19 ± 4.55	14.95 ± 3.28	0.76 (5.37)	12.70 ± 4.28	13.13 ± 4.16	0.43 (3.42)	0.150
Basal metabolic rate (kcal)	1329.52 ± 121.05	1328.48 ± 122.37	−1.05 (−0.08)	1322.70 ± 146.55	1322.35 ± 154.41	−0.35 (−0.03)	0.921
Fitness score	62.19 ± 5.60	61.57 ± 5.87	−0.62 (−1.00)	64.78 ± 6.95	63.96 ± 6.69	−0.83 (−1.28)	0.194
Systolic blood pressure (mmHg)	107.21 ± 12.55	108.62 ± 12.01	1.41 (1.31)	109.57 ± 10.30	108.98 ± 10.04	−0.58 (−0.53)	0.407
Diastolic blood pressure (mmHg)	72.90 ± 6.38	71.06 ± 5.99	−1.84 (−2.53)	73.90 ± 9.25	69.73 ± 6.44 *	−4.17 (−5.64)	0.871
Pulse rate (/min)	81.30 ± 11.02	82.98 ± 12.76	1.68 (2.07)	75.78 ± 8.77	77.18 ± 10.87	1.40 (1.85)	0.215
Respiratory rate (/min)	18.29 ± 2.35	18.86 ± 1.49	0.57 (3.13)	18.52 ± 2.59	18.96 ± 1.33	0.43 (2.35)	0.929
Body temperature (°C)	36.36 ± 0.36	36.40 ± 0.23	0.05 (0.13)	36.07 ± 0.55	36.45 ± 0.26 *	0.39 (1.07)	0.683
Hemoglobin saturation (%)	97.71 ± 0.85	97.90 ± 0.83	0.19 (0.19)	97.78 ± 1.00	98.26 ± 0.81 *	0.48 (0.49)	0.416
Physical activity (kcal/day)	1408.82 ± 157.89	1394.15 ± 150.43	−14.67 (−1.04)	1406.26 ± 77.21	1402.50 ± 71.03	−3.76 (−0.27)	0.659
Dietary intake (kcal/day)	1425.37 ± 54.36	1426.11 ± 40.63	0.74 (0.05)	1407.31 ± 82.77	1400.06 ± 72.03	−7.25 (−0.52)	0.107

Data are presented as mean ± standard deviation (SD); total *n* = 44. * *p* < 0.05 vs. before supplementation.

**Table 2:** Biochemical parameters of participants in the placebo and asparagus groups at baseline and after 12 weeks of supplementation.

Parameters	Placebo Group (*n* = 21)			Asparagus Group (*n* = 23)			*p*-Value (After vs. After)
	Before	After	Δ Change (%)	Before	After	Δ Change (%)	
FBG (mg/dL)	97.38 ± 11.04	99.90 ± 12.17	2.52 (2.59)	94.52 ± 10.83	92.27 ± 8.32 **	−2.25 (−2.38)	0.045
Insulin (µU/mL)	12.31 ± 6.18	19.17 ± 9.76 *	6.86 (55.74)	14.82 ± 11.50	16.89 ± 9.75	2.07 (13.94)	0.271
HOMA-IR	2.96 ± 1.48	4.71 ± 2.26 *	1.75 (59.17)	3.46 ± 2.77	3.85 ± 2.24	0.40 (11.45)	0.143
HOMA-B	7.80 ± 4.68	11.46 ± 7.79 *	3.65 (46.80)	11.04 ± 9.87	12.44 ± 8.17	1.40 (12.65)	0.950
TC (mg/dL)	203.00 ± 34.13	206.29 ± 23.47	3.29 (1.62)	200.61 ± 44.21	202.86 ± 41.16	2.25 (1.12)	0.612
TG (mg/dL)	138.76 ± 85.25	181.33 ± 128.58 *	42.57 (30.68)	116.26 ± 72.98	113.23 ± 54.69 **	−3.03 (−2.61)	0.043
LDL-C (mg/dL)	136.91 ± 47.36	129.64 ± 44.12	−7.28 (−5.31)	141.48 ± 37.87	129.52 ± 28.35 *	−11.95 (−8.45)	0.891
HDL-C (mg/dL)	50.10 ± 9.71	49.10 ± 12.44	−1.00 (−2.00)	53.13 ± 11.53	53.68 ± 15.48	0.55 (1.04)	0.538
TC/HDL-C ratio	4.22 ± 1.20	4.46 ± 1.22 *	0.24 (5.76)	3.99 ± 1.38	4.07 ± 1.41	0.08 (2.01)	0.499
TG/HDL-C ratio	3.02 ± 2.19	4.26 ± 3.63 *	1.23 (40.81)	2.42 ± 1.71	2.42 ± 1.55 **	0.01 (0.22)	0.032
LDL-C/HDL-C ratio	2.96 ± 1.15	2.85 ± 1.07	−0.11 (−3.81)	2.78 ± 1.32	2.68 ± 1.32	−0.10 (−3.59)	0.746

Data are presented as mean ± standard deviation (SD); total n = 44. FBG, fasting blood glucose; HDL-C, high-density lipoprotein cholesterol; HOMA-B, homeostasis model assessment of β-cell function; HOMA-IR, homeostasis model assessment of insulin resistance; LDL-C, low-density lipoprotein cholesterol; TC, total cholesterol; TG, triglycerides. * *p* < 0.05 vs. baseline; ** *p* < 0.05 vs. placebo group.

## Data Availability

The data are available upon request from the corresponding author. Restrictions apply to the availability of these data due to privacy and ethical considerations.

## Abbreviations

The following abbreviations are used in this manuscript:

ANOVA	Analysis of variance
AUC	Area under the curve
BG	Blood glucose
BM	Body mass
BMI	Body mass index
BP	Blood pressure
DBP	Diastolic blood pressure
FBG	Fasting blood glucose
GC-MS	Gas chromatography–mass spectrometry
GSH	Glutathione
HDL-C	High-density lipoprotein cholesterol
HOMA-B	Homeostasis model assessment of β-cell function
HOMA-IR	Homeostasis model assessment of insulin resistance
IL	Interleukin
LDL-C	Low-density lipoprotein cholesterol
MDA	Malondialdehyde
OGTT	Oral glucose tolerance test
ROS	Reactive oxygen species
SBP	Systolic blood pressure
SOD	Superoxide dismutase
TC	Total cholesterol
TG	Triglyceride
TNF-α	Tumor necrosis factor-alpha
W/H	Waist-to-hip
